# Effect of Antifreeze Glycoproteins on Organoid Survival during and after Hypothermic Storage

**DOI:** 10.3390/biom9030110

**Published:** 2019-03-19

**Authors:** Guizela Huelsz-Prince, Arthur L. DeVries, Huib J. Bakker, Jeroen S. van Zon, Konrad Meister

**Affiliations:** 1 AMOLF, Science Park 104, 1098 XG Amsterdam, The Netherlands; g.huelsz@amolf.nl (G.H.-P.); h.bakker@amolf.nl (H.J.B.); J.v.Zon@amolf.nl (J.S.v.Z.); 2 University of Illinois at Urbana-Champaign, Urbana, IL 61801, USA; adevries@life.illinois.edu; 3 Max Planck Institute for Polymer Research, Ackermanweg 10, D-55128 Mainz, Germany

**Keywords:** antifreeze glycoproteins, organoids, hypothermic storage, fluorescence microscopy

## Abstract

We study the effect of antifreeze glycoproteins (AFGPs) on the survival of organoids under hypothermic conditions. We find that the survival of organoids in cold conditions depends on their developmental stage. Mature organoids die within 24 h when being stored at 4 °C, while cystic organoids can survive up to 48 h. We find that in the presence of AFGPs, the organoid survival is prolonged up to 72 h, irrespective of their developmental stage. Fluorescence microscopy experiments reveal that the AFGPs predominately localize at the cell surface and cover the cell membranes. Our findings support a mechanism in which the positive effect of AFGPs on cell survival during hypothermic storage involves the direct interaction of AFGPs with the cell membrane. Our research highlights organoids as an attractive multicellular model system for studying the action of AFGPs that bridges the gap between single-cell and whole-organ studies.

## 1. Introduction

Hypothermic preservation is a commonly used method in which cells, tissues, or organs are maintained at low temperatures (1–10 °C) for short-term storage situations that enable distant transport. During hypothermic storage, cells encounter cold stress that affects their cell physiology, metabolic activity, and regulation of ion equilibration across membranes [1]. Much effort has been made in the optimization of storage solutions that minimize cold-induced damage and that increase the time interval that cells and organs can be cold-stored [2]. However, up to now, this time interval remains limited, which significantly restricts the donor organ supply, access, and utility. Numerous organisms have evolved adaptive mechanisms for their survival in fluctuating cold temperatures and icy environments [3]. Usually, these mechanisms involve the production of antifreeze proteins (AFPs) and antifreeze glycoproteins (AFGPs) [3,4]. AF(G)Ps have the unique abilities to inhibit ice recrystallization, dynamically shape ice crystals, and depress the freezing point of a solution in a noncolligative manner [5,6]. In addition, several AF(G)Ps have shown promising results in applications that include the enhanced hypothermic storage of cold-sensitive cells, embryos, and other biological tissues [7,8,9,10]. Rubinsky et al. showed that the fertility of bovine oocytes [11] could be significantly improved by the addition of fish AF(G)Ps, and that rat livers can be stabilized at low temperatures using AF(G)Ps [12]. The effects of AF(G)Ps on hypothermic storage and cryopreservation have, however, been discordant, and some studies reported negligible and even adverse effects [13]. Moreover, the mechanism(s) by which AF(G)Ps may exert a protective effect on cells during cold storage is not understood, and successful cold storage of multicellular system, in particular organs, is still out of reach.

So far, the mechanism(s) by which AF(G)Ps exert a protective action of AF(G)Ps is studied in vivo, mostly in single cells using 2D cell culture [9]. Studies on multicellular systems are typically performed on whole embryos or organs [12,14], whose large sizes pose severe challenges in terms of the amount of AFPs required and imaging the action of AFP on the cellular and molecular level.

Organoids are self-organizing three-dimensional structures that are grown from stem cells in vitro, which recapitulate the essential features of organ architecture and function [15]. Organoids bridge the gap between traditional 2D cell cultures and organs, since they are stable cultures that allow for differentiation, cell–cell and cell–matrix interactions, and 3D organization; yet they remain affordable and easily accessible for manipulation and experimentation. Since their introduction nearly a decade ago, organoids from several different tissues have been developed, starting a revolution in both the biological and medical fields. Organoids provide previously inaccessible insights into morphogenesis, stem cell biology, and disease, and hold great promise for the future of personalized medicine [16,17].

Here, we study the survival of intestinal organoids under hypothermic conditions, and how this survival is affected by the addition of AFGPs. We observe that AFGPs have a clear life-prolonging effect on organoids in all different developmental stages.

## 2. Materials and Methods

### 2.1. AFGP Purification

Antifreeze glycoproteins were purified from the Antarctic toothfish *Dissostichus mawsoni* and fluorescently labeled using fluorescein isothiocyanate (FITC) as described previously [18].

### 2.2. Organoid Culture

Intestinal organoids isolated from C57BL/6 mice were a gift from Norman Sachs (Hubrecht Institute, The Netherlands). Organoids were cultured according to previously described protocols with minor adjustments [19]. In short, organoids were embedded in basement membrane extract (BME, Trevingen, Gaithersburg, USA) droplets and overlaid with IntestiCult Organoid Growth Medium (STEMCELL Technologies, Vancouver, Canada), which was changed every two to three days. Organoid passaging was performed by mechanically dissociating crypts using a narrowed glass pipette.

### 2.3. Cold Storage Experiements

Cold storage experiments were performed with organoids that were embedded in a mixture of two-thirds BME and one-third 0.5% agarose (VWR Chemicals, Radnor, USA). We added agarose to prevent BME droplets from disintegrating, as BME becomes liquid at temperatures ~4 °C. The BME/agarose mixture supported organoid growth, and normal development was observed for up to three passages. The cold storage experiments were started two to three days after plating the organoids in the BME/agarose mixture. The plates were sealed with Parafilm and placed in an air-tight bag containing 5% CO_2_ and moved to 4 °C. Organoids were stored in cold conditions for different time periods, after which the plates were returned to an incubator set at 37 °C. The color of the medium was assessed throughout the procedure to ensure normal pH values and that a lack of CO_2_ was not affecting the organoid viability.

In the survival experiments, AFGP and α-lactalbumin were added to the medium one hour before the organoids were transferred to a 4 °C fridge. The concentration of the added proteins in the medium was 10 mg/mL [9]. The experimental design is outlined in Supplementary Figure S1. For each condition, individual plates with organoids were prepared, and each plate was left at 4 °C for different periods of time ranging from 24 to 120 h. During hypothermic storage, individual organoids were followed and visually assessed every 24 h to determine their viability. At each time point, an organoid was classified as alive or dead. Dead organoids were easily recognizable by an overall lack of structure and the presence of dark debris where the organoid was previously located, as shown in Supplementary Figure S2. Data were collected from the number of independent experiments presented in Supplementary Figure S1. After cold storage, the organoids were returned to the 37 °C incubator and their growth and viability were assessed again after 2–3 days. Living organoids grew considerably, while dead debris remained the same. Hence, the visual distinction between alive and dead organoids was straightforward.

### 2.4. Fluorescein Diacetate Test

Viability tests using fluorescein diacetate (FDA) were performed to test the accuracy of the visual viability assessment [20].

FDA was added to the medium at a concentration of 10 µg/mL, and the medium was removed after 5 min and replaced with phosphate-buffered saline. After 15 min, the wells were washed twice, and the organoids were imaged using a widefield fluorescence microscope (Axio Vert.A1, Zeiss). FDA tests were not used to completely quantify organoid viability, due to an observed inability of the FDA to completely penetrate BME droplets. The dye showed false negatives when inspecting organoids that were deeply embedded in the BME/agarose gel, either because the FDA was unable to fully penetrate the gel, or because it was taken up by the organoids in the periphery of the gel more rapidly than it could reach the more deeply embedded ones. As a result, fluorescence could only be observed in healthy organoids close to the edges of the droplets, while live organoids located in central regions did not show any fluorescence that could be distinguished from the background. The visual assessment of organoids in the periphery of the gel coincided with the FDA staining, which supports the validity of our visual protocol.

### 2.5. Imaging of FITC-AFGP

Organoids were mechanically dissociated, embedded in BME, and plated in 8-well imaging chambers. Two to three days after passaging, 0.5 mg/mL of FITC-AFGP was added to the medium and, after one hour, the plates were imaged using a scanning confocal microscope (Eclipse Ti; Nikon).

### 2.6. Statistical Analysis

**Figure 1:**
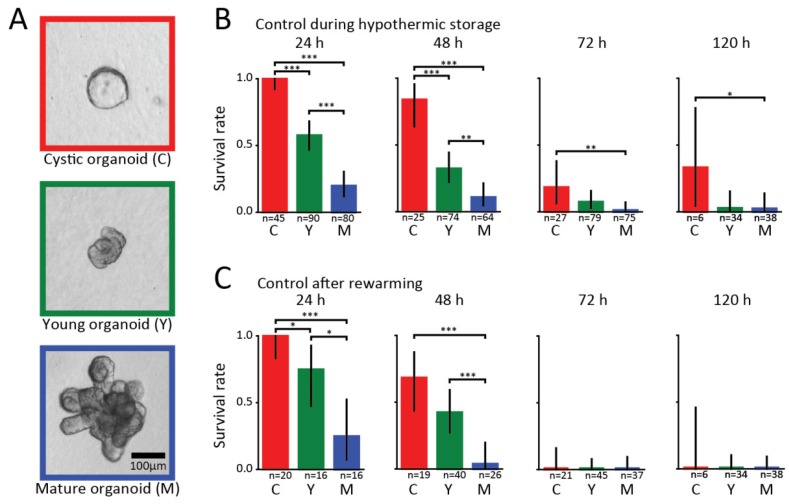
Survival of organoids under hypothermic conditions with no added proteins. (**a**) Classification of organoids according to their developmental stage. Cystic organoids (left panel, red) are spherical structures with a large lumen and thin walls. Young organoids are no longer spherical, have a small lumen, thick walls, and small buds rather than crypts (middle panel, green). Mature organoids clearly have grown crypt structures (right panel, blue). (**b**) Survival rates of organoids at different developmental stages obtained from visually assessing single cells every 24 h during hypothermic storage periods ranging from 24 to 120 h. Bars show results pooled from different independent experiments. (**c**) Corresponding survival rates obtained from a visual assessment performed two to three days after the organoids were returned to the incubator. For each organoid class and experimental condition, the number (n) of organoids examined is indicated. Asterisks denote significant differences (* *p* < 0.05, ** *p* < 0.01, *** *p* < 0.001).

**Figure 2:**
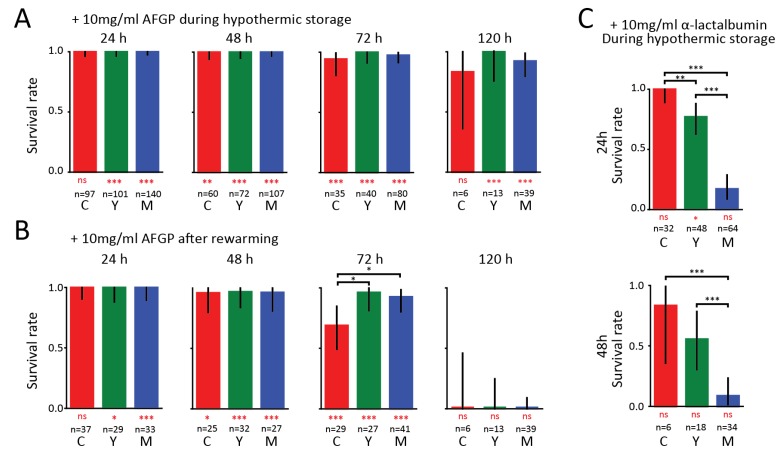
Survival of organoids under hypothermic conditions with antifreeze glycoprotein (AFGP) and α-lactalbumin present. (**a**) Survival rates of organoids at the different developmental stages when AFGP was added to the growth medium. Rates were obtained from visually assessing the organoids every 24 h during hypothermic storage periods ranging from 24 to 120 h. Bars show results pooled from different independent experiments. (**b**) Corresponding survival rates obtained from a visual assessment performed two to three days after the organoids were returned to the incubator. (**c**) Survival rates of organoids at the different developmental stages when α-lactalbumin was added to the growth medium. Black asterisks denote significant differences between developmental stages, and red asterisks below bars denote differences from controls in Figure 1 (* *p* < 0.05, ** *p* < 0.01, *** *p* < 0.001, ns: not significant).

We performed a statistical analysis of the data using the two-sided Fisher’s exact test, *p* < 0.05 was considered statistically significant. In all plots (Figure 1B,C, Figure 2), the error bars represent 95% Clopper–Pearson confidence intervals. In cases where we performed multiple independent experiments, the data were pooled and plotted together.

## 3. Results

We investigated the survival rate of intestinal organoids under hypothermic conditions at time periods spanning from 24 to 120 h. The organoids were categorized into cystic, young, or mature, which correspond to different developmental stages. Cystic organoids are spherical structures consisting of stretched-out undifferentiated cells forming a large lumen with thin walls (left panel of Figure 1a). Young organoids are no longer spherical, have a small lumen, thick walls, and small buds without pronounced crypts, as shown in the middle panel of Figure 1a. Mature organoids possess clearly visible crypt structures as shown in the right panel of Figure 1a. Every 24 h, we took a selection of organoids for which we performed a visual assessment of their viability. Subsequently, the assessed organoids were returned in the incubator and evaluated on how they recovered from the hypothermic exposure. We observed considerable growth of live organoids after they were being rewarmed, and we used this property to distinguish between the live and dead organoids. In fact, we also observed that healthy-looking crypts were able to bud from such organoids, suggesting that stem cells are present, which would allow for organoid passaging.

The visual cell viability assessment was supported by fluorescein diacetate fluorescence within the cytoplasm. FDA serves as a viability probe that measures enzymatic activity, which is obligatory to activate its fluorescence, and cell membrane integrity, which is necessary for intracellular retention of the fluorescent product. FDA was chosen due to its low toxicity, which allows for an in situ viability assessment throughout the timespan of the experiments. The use of live/dead stains is not straightforward for organoids as the lumen of healthy organoids usually contains dead cell material because old cells are shed into the lumen, mimicking the cell shedding that occurs at the tips of the villi in the intestine. As a result, stains that target dead cells are not appropriate as they would show false positives.

In Figure 1B, we present the results of the survival of the hypothermic organoids without added proteins. We find that after 24 h of exposure to 4 °C, 100% of the cystic and 58% of the young organoids survived, while only 20% of the mature ones survived. After 48 h of exposure, we find that 84% of cystic organoids were still alive, and the rate dropped to 32% and 11% for young and mature organoids, respectively. At 72 and 120 h, the survival rates decreased dramatically, with a maximum of 33% for cystic and 8% for young and mature organoids. Low variability between independent experiments was observed (Supplementary Figure S3).

In Figure 1C, we show the results of organoid survival after we returned them to the incubator at 37 °C. In agreement with the visual inspection results shown in Figure 1B, we find that most cystic organoids survived hypothermic exposures of up to 48 h, and could readily be brought back into culturing. By contrast, a significant portion of the larger organoids did not survive rewarming and the return to the incubator. We further observe that no organoids survived rewarming after being exposed to hypothermic storage periods of 72 h or longer.

### Antifreeze Glycoproteins

In order to evaluate whether the addition of AFGP influences organoid survival under hypothermic conditions, we repeated the previous experiments after adding AFGP to the growth medium. For cold-storage experiments, an AFGP concentration of 10 mg/mL was chosen, as this concentration showed protective effects in a previous study on cells [9]. We also performed control experiments in which we added the non-antifreeze protein α-lactalbumin to the growth medium. We chose α-lactalbumin as the control protein as this protein has a similar size, shows no antifreeze activity, and albumins have been used as control proteins in previous AF(G)P studies [9].

In Figure 2, we present the survival rates of organoids at different developmental stages in the presence of AFGP and α-lactalbumin. After 24 and 48 h, we find that 100% of the organoids appeared alive in the presence of AFGPs (Figure 2a). At 72 and 120 h, the survival rates remained above 80% for cystic and above 90% for young and mature organoids. We thus find that the addition of AFGP to the medium has a beneficial effect on organoid survival at all developmental stages. We also observed low variability between independent experiments (Supplementary Figure S3).

Upon return to the incubator (Figure 2B), 100% of organoids were alive after hypothermic storage of 24 h. The positive effect of AFGP on the viability of the organoids was also observed after 48 h where 96% of organoids survived. After 72 h, the survival rates remained high for young and mature organoids (~93%), while the rate dropped to 69% for cystic organoids. Even after remaining in hypothermic storage for 120 h, most organoids survive. However, none of the organoids that were hypothermically stored for 120 h survived after being warmed up in the incubator. In strong contrast to AFGP, α-lactalbumin did not provide any protection against hypothermic stress, as shown in Figure 2C. We find that after 24 h of cold exposure 100% of cystic, 77% of young and 17% of mature organoids survived. After 48 h, 83% of cystic and 56% of young organoids are still alive, but only 9% of mature organoids survived. The survival rates in the presence of α-lactalbumin are comparable to the survival rates observed when no proteins are added (Figure 1b).

**Figure 3:**
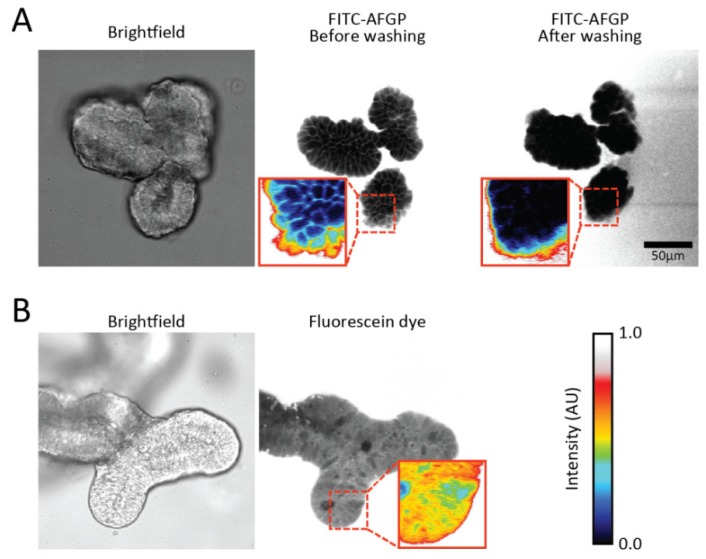
Fluorescence images of organoids in the presence of FITC-labeled AFGP. (**a**) Brightfield and fluorescence images of an organoid with FITC-AFGP in the medium reveal that fluorescence is observed on the cell outlines. Washing of the imaging wells with buffer eliminated the fluorescence pattern; (**b**) Brightfield and fluorescence images of an organoid with fluorescein diacetate in the medium show that fluorescence is present in the cytoplasm of most cells. Pixel intensities are colored in insets to highlight the observed fluorescence patterns. Colors represent the same intensities in all images.

Figure 3 shows fluorescence images of organoids in the presence of FITC-AFGP. We find that the labeled AFGPs are predominately localized on the cell surface. This finding suggests that AFGPs preferably localize at cell membranes. Control experiments using solely fluorescein dye showed no fluorescent pattern (Figure 3b), indicating that AFGP (and not the fluorescent label) was responsible for the membrane localization. We observe that washing the organoids with PBS buffer resulted in disappearance of the observed fluorescent pattern, as shown in Figure 3a. This observation suggests that AFGPs were only loosely bound to the membrane and were not inserted into the membrane.

## 4. Discussion

The hypothermic storage of cells causes cold stress that affects the cell physiology, metabolic activity, and regulation of ion equilibration across membranes [1]. We observed a statistically significant dependence of organoid survival after hypothermic storage on the developmental stage of the organoid. More developed mature organoids showed much lower viability rates compared to cystic organoids. Hypothermic storage likely activates different sets of stress pathways that are cell- and tissue-specific. Cystic organoids are composed of cells that strongly differ from the cells of mature organoids. Cystic organoids, sometimes referred to as “enterospheres”, consist of undifferentiated cells with stem cell-like properties. They differ from mature intestinal stem cells in having a distinct set of gene expression patterns as well as different sets of active signaling pathways [21,22,23]. These characteristics enable cystic organoids to recapitulate the essential features of intestinal tissue under a state of repair after injury [21,22]. By contrast, mature organoids have only a small percentage of stem and progenitor cells, and are mostly composed of fully differentiated secretory and absorptive cells which resemble the intestinal tissue in a homeostatic state. The distinct cell identities and functions in both cystic and mature organoids likely result in differences in their ability to respond to stress during and after hypothermic storage. Young organoids are likely to contain subsets of both cystic and mature properties, thus explaining their intermediate survival rates.

The characteristic shape and behavior of cystic organoids (a round and enlarged lumen, thin walls formed by stretched-out cells, and occasional rapid contractions observed after expulsion of material from the lumen (data not shown) suggest that they are under high intraluminal pressure due to accumulation of fluid within the lumen. This cystic morphology has been documented when organoids swell as a result of adding forskolin [24] or cholera toxin [25] to the medium. Furthermore, addition of the signaling factor Wnt3a resulted in organoids adopting a cystic morphology as well as their cells assuming an undifferentiated state [26]. In all cases, the cystic organoid morphology has been linked to increased chloride secretion by the cystic fibrosis transmembrane conductance regulator (CFTR) ion channel which, in turn, causes increased fluid secretion into the lumen. This suggests that ion transportation in cystic organoids is highly dynamic compared to more mature organoids. We speculate that as a result, ion leakage caused by hypothermic storage has a smaller impact on cystic organoids in comparison to young and mature organoids, accounting for their higher survival rate.

We find that the addition of AFGPs to the medium has a statistically significant positive effect on the cold survival of organoids, which is in line with previous studies [10]. Current mechanisms that explain the positive effect of AFPs on the cold survival of cells involve the blockage or alteration of the flow of ions into cells [27,28] and the protection of cell membranes as they pass through their phase transition temperatures [29,30]. Tomczak et al. proposed that AFGPs may form a monolayer covering the membrane surface, thereby reducing the leakage of ions across the membrane as it is cooled through its thermal transition temperature [30]. Using fluorescence microscopy, we find strong evidence that AFGPs localize at the cell membranes, suggesting that the protection mechanism of AFGPs is indeed closely connected to their interaction with cell membranes. From the obtained fluorescence data, we cannot infer whether AFGPs target specific ion channels. We observe that AFGPs loosely interact with the cell membranes of the organoids and find that AFGPs do not only cover model membranes but entire multicell systems.

Interestingly, we further observe that upon rewarming, the survival rate remains high (93%) for young and mature organoids while it drops to 69% for cystic organoids in the presence of AFGPs. Garner et al. showed that AFGPs interact with a model membrane at both 5 and 30 °C, but that the interaction at 30 °C is much weaker [31]. We speculate that the distinct cell identities in cystic and more mature organoids do not only affect their ability to respond to stress during hypothermic storage but also to stress experienced during rewarming. More mature organoids could, for instance, have a different membrane composition with more phosphate groups. Such groups could enable a better interaction with AFGPs and a prolonged protection. Clearly, the response to rewarming stress must also be considered when investigating potential AFGP applications for the storage of cells and tissues in the cold for medical purposes.

## 5. Conclusions

In conclusion, organoids provide a flexible and previously inaccessible method of studying the effects of cryopreservation on multicellular systems, which could bring us a step closer to the cryopreservation of entire organs. We find clear evidence that AFGPs have a strong positive effect on the survival of organoids during and after hypothermic storage at 4 °C. If this protection against hypothermia-related perturbations could be extended for even longer periods and to complete intestinal organs, then it would have enormous practical implications for the transfer and storage of organs.

## Supplementary Materials

The following are available online at https://www.mdpi.com/2218-273X/9/3/110/s1, Figure S1: Design of the Hypothermic storage experiments. Figure S2: Optical images of live and dead organoids. Figure S3: Average survival rates from independent organoid experiments.
