# Supplementary material for: Effect of Antifreeze Glycoproteins on Organoid Survival during and after Hypothermic Storage
Source: Biomolecules. 2019 Mar 19;9(3):110. doi: 10.3390/biom9030110 (PMC6468685; doi:10.3390/biom9030110)
Supplement: Supplementary file 1 [file biomolecules-09-00110-s001.pdf]

Supplementary Information:

Effect of Antifreeze Glycoproteins on Organoid Survival During and After Hypothermic Storage

Authors: G. Huelsz-Prince[1], A. L. DeVries[2], Huib J. Bakker[1], J. S. van Zon[1] & K. Meister[3]

Affiliations:

[1]AMOLF, Science Park 104, 1098 XG Amsterdam, The Netherlands

[2]Department of Animal Biology, University of Illinois, Urbana, Illinois 61801, United States

[3]Max Planck Institute for Polymer Research, Ackermannweg 10, D-55128 Mainz, Germany

*Correspondence to: meisterk@mpip-mainz.mpg.de

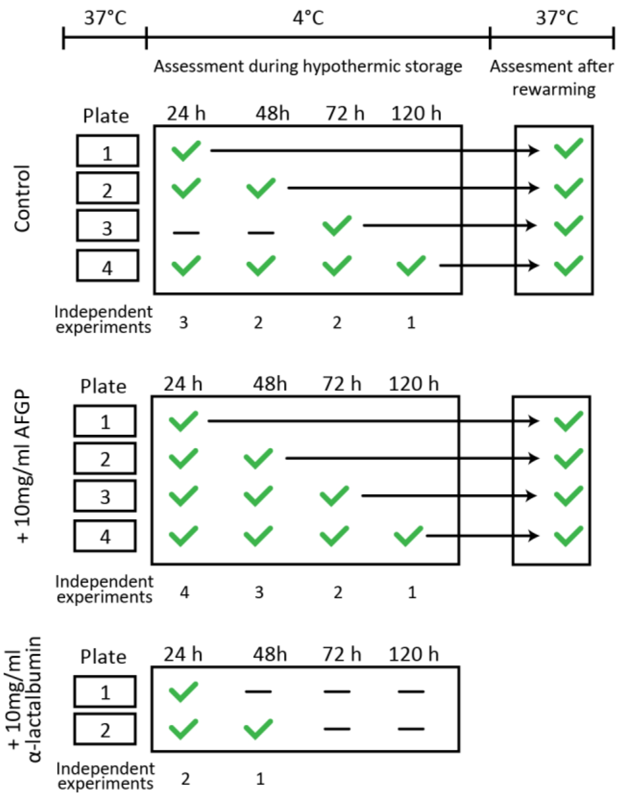

Figure S1. Design of the Hypothermic storage experiments. For each of the three conditions tested, different numbers of plates with organoids were prepared. Each plate was placed at 4°C for different periods of time during which organoid viability was assessed every 24 hours (except for control plate 3). After hypothermic storage, plates were returned to the incubator at 37°C and organoid viability was assessed again. Since assessment during hypothermic storage was performed on different plates every 24 hours, for most time points data was collected from a number of independent experiments.

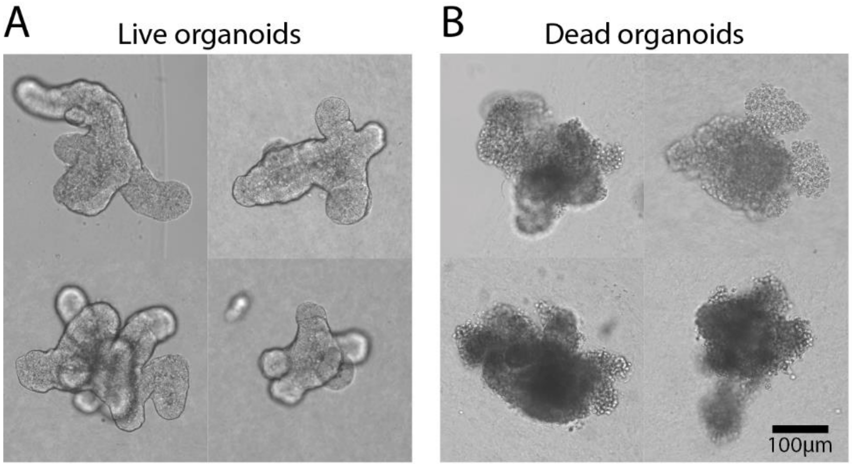

Figure S2. Optical images of live and dead organoids Dead organoids are easily recognizable by an overall lack of structure and the presence of dark debris where the organoids was located.

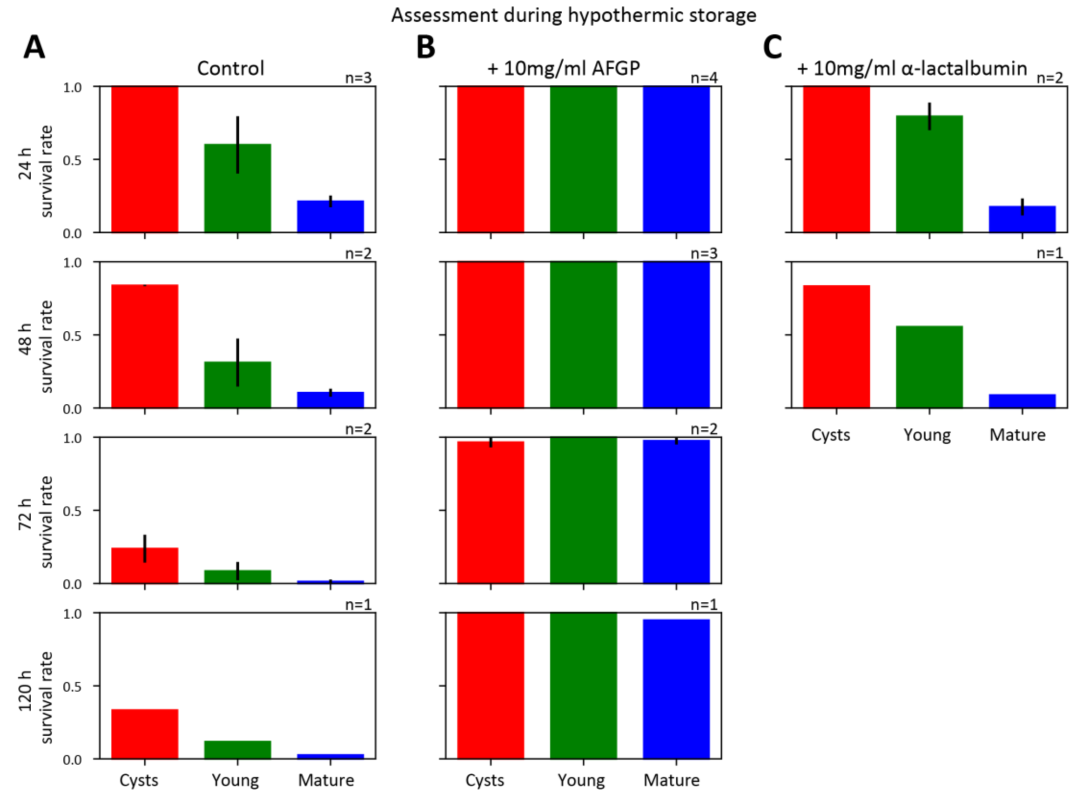

Figure S3. Average survival rates from independent organoid experiments. Average survival rates were obtained when assessing the organoid viability during hypothermic storage on different plates. Error bars represent the standard deviation. The number of independent experiments (in accordance to Figure S1) is shown.
